# Pineal region tumours in the sitting position: how I do it

**DOI:** 10.1007/s00701-021-04821-3

**Published:** 2021-05-02

**Authors:** Priya Sharma, Mohd Abdul, Manprit Waraich, George Samandouras

**Affiliations:** 1 Imperial College School of Medicine, London, UK; 2 Victor Horsley Department of Neurosurgery, The National Hospital for Neurology and Neurosurgery, Queen Square, London, UK; 3 Department of Neuroanaesthesia, The National Hospital for Neurology and Neurosurgery, Queen Square, London, UK; 4 UCL Queen Square Institute of Neurology, University College London, Queen Square, London, UK

**Keywords:** Pineal tumours, Sitting position, Air-embolism, Pineoblastoma, Germ-cell tumours

## Abstract

**Background:**

Pineal region tumours remain challenging neurosurgical pathologies.

**Methods:**

Detailed anatomical knowledge of the posterior incisural space and its variations is critical. An opaque arachnoidal membrane seals the internal cerebral and basal veins, leading to thalamic, basal ganglia, mesencephalic/pontine infarctions if injured. Medium-size tumours can be removed en-bloc with all traction/manipulation applied on the tumour side, virtually without contact of ependymal surfaces of the pulvinars or third ventricle. Sacrifice of the cerebello-mesencephalic fissure vein may be required.

**Conclusions:**

The sitting position offers superior anatomical orientation and remains safe with experienced teams. Meticulous microsurgical techniques and detailed anatomical knowledge are likely to secure safe outcomes.

**Supplementary Information:**

The online version contains supplementary material available at 10.1007/s00701-021-04821-3.

## Relevant surgical anatomy

The complex pineal region can be accessed by the supracerebellar/infratentorial (SC/IT) approach, occipital transtentorial, posterior interhemispheric transcallosal and the combined supra-/IT approach, for giant tumours. Although off midline (lateral and far-lateral) approaches are available for pineal region tumours, the midline IT/SC approach offers optimal surgical orientation in the region of posterior incisura, bounded superiorly by the inferior aspect of the splenium, the forniceal crura and the hippocampal commissure and inferiorly by the vermian culmen, and medially and laterally, by the central and quadrangular lobules, respectively [7]. The posterior incicura extends inferior to the cerebellomesencephalic fissure that accomodates its corresponding vein, also termed precentral vein, a key, midline venous landmark *(*Fig. 4b, c*)*.

### Vascular anatomy

The second (P2) and third (P3) segments of the posterior cerebral artery (PCA) lie between the thalamic pulvinar and superior colliculi, while loops of the superior cerebellar artery (SCA) are often visible anterior to pineal gland. The surgical venous anatomy of the pineal region is complex and are covered by an arachnoid layer of varied thickness, ranging from thick and opaque to thin and translucent (Fig. 4b). The vein of Galen (VG) is the most dorsal part of the venous complex, joining the straight sinus (SS) while the paramedian, paired basal veins of Rosenthal (BvRo) exit the ambient cistern and converge to the VG. The internal cerebral veins (ICv) lie deeper, laterally and slightly underneath the BvRo exiting through the cistern of the velum interpositum, also converging to the VG [4].

### Cisternal anatomy

**Fig. 1:**
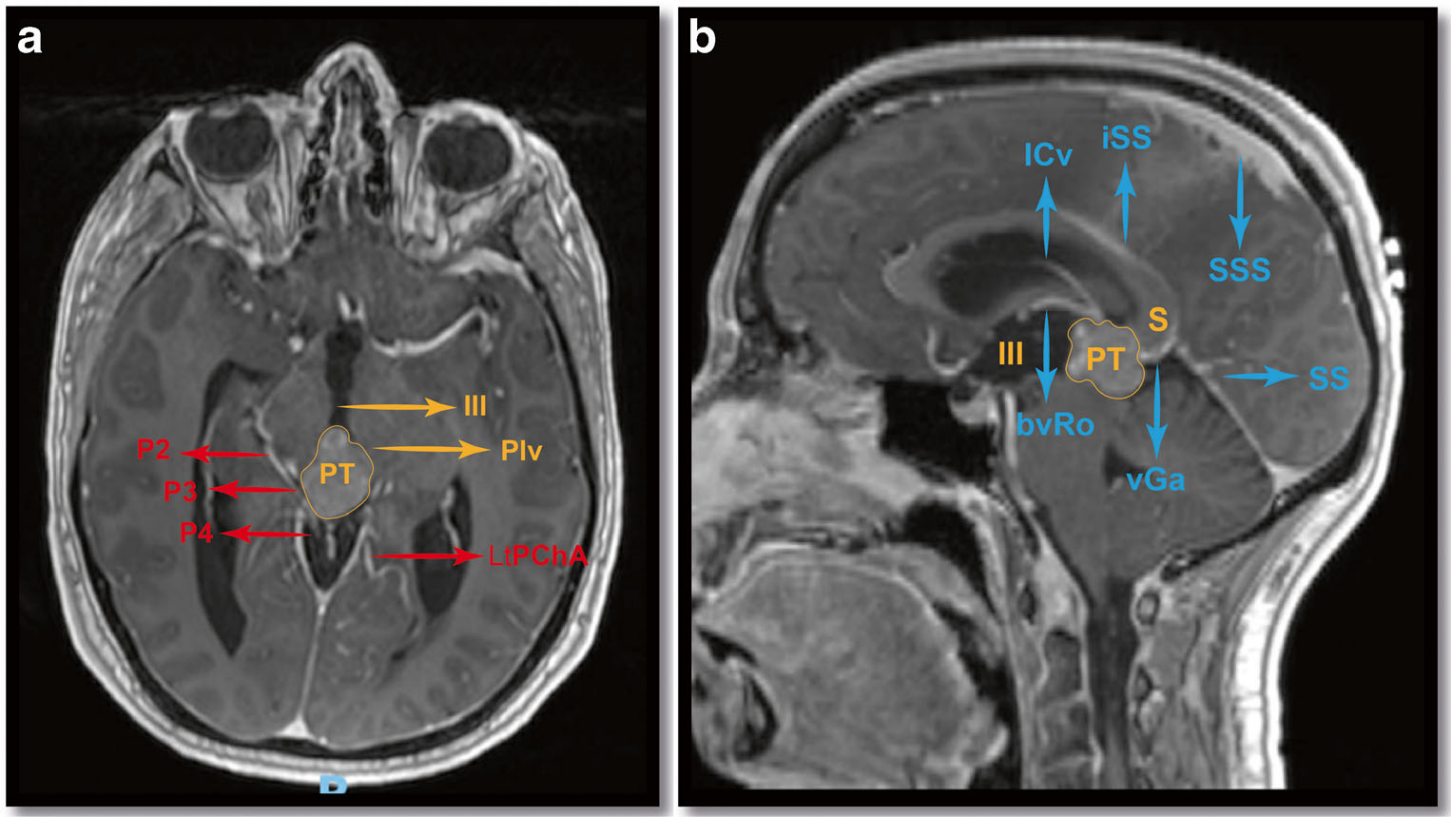
Imaging and surgical anatomy features of the pineal region. Axial (**a**) and sagittal (**b**) views of a pineal region tumour, with key arterial and venous structures, respectively. PT, pineal tumour; Plv, pulvinar of thalamus; III, 3rd ventricle; P2, P3, P4, second, third and fourth segments of the posterior cerebral artery; LtPChA, lateral posterior choroidal artery; ICv, inferior cerebral vein; BvRo, basal vein of Rosenthal; vGa, vein of Galen; SS, straight sinus; iSS, inferior sagittal sinus; SS, straight sinus; SSS, superior sagittal sinus; S, splenium of corpus callosum

The unpaired quadrigeminal cistern contains all three major venous systems, VG, the confluences of both BvRo and ICvs, P4 and the posterior medial choroidal artery. The paired ambient cisterns contain P2 and P3, the anterior choroidal artery, the CvRo and SCA segments. The cistern of the velum interpositum, extending from the foramen of Monroe anteriorly to the habenula encloses the ICvs and the posterior medial choroidal artery. The quadrigeminal and velum interpositum cisterns communicate with the posterior pericallosal cistern [4] (Fig. 1).

## Description of the technique

### Preoperative work-up

The SC/IT approach in the sitting position (SP) offers optimal surgical field without cerebellar retraction but is fraught with the most feared systematic complication, venous air embolism [2, 3] (Tables 1 and 2). Reported incidence of VAE varies significantly as accurate diagnostic criteria are not standardised. In addition, not all transoesophageal echo-detected VAE are clinically significant (Table 1). A bubble echo can exclude patent foramen ovale, detected in 30% of population, a SP contraindication.

**Table 1:** Clinical signs in relation to volume of embolised air

Clinical signs and volume of air	
Small < 0.5 mL/kg	
Sudden decrease in EtCO2 by ≥ 2 mmHg [8] Increase in PaCO2 [8] Difference between EtCO2 and PaCO2 > 10 mmHg [6] Increase in EtN2 > 0.04% [8] Hypoxia [8]	
Moderate 0.5–2.0 mL/kg	
Jugular venous distension [8, 9] Unexplained hypotension [8, 9] Hypoxia [8, 9] ECG: tachyarrhythmias [8], right heart strain pattern (peaked P wave, RBBB, right axis deviation) [9, 10] or acute ischaemia ST changes [8–10] Cerebral ischaemia (changes in mental status postoperatively) [8, 10] Pulmonary hypertension detected via pulmonary artery catheter [6] Pulmonary oedema [9, 10]	
Large > 2.0 mL/kg	
‘Water-wheel’ or ‘mill-wheel’ murmur [8, 10] Acute-onset right-sided heart failure [8, 9] Cardiovascular collapse [8, 9] Cardiac arrest [9] Fatal at 3–4 mL/kg [8, 10]

**Table 2:** Advantages and disadvantages of the sitting position in neurosurgery

Advantages	Disadvantages
Operative/positional	
Gravity facilitates culmen/cerebellar descent and tumour dissection from splenium Facilitates drainage of blood, clear operative field Better anatomical orientation of a complex neurovascular region Natural corridor with no normal tissue violation during approach	Fatigue of operator’s hands Risk of postoperative haematoma when patient returns to horizontal position (0.4%) Risk of brachial plexus injury if arms allowed at sides rather than folded on abdomen [12] Cervical quadriplegia has been reported (neck flexion + anterior spinal artery compression + systemic hypotension) [12] Sciatic nerve injury from nerve tension (piriformis syndrome) prevented by gentle knee flexion [12]
Anaesthetic	
Easy ventilation (unencumbered chest)	Risk of VAE (%) Invasive anaesthetic monitoring with CVP catheter (risk of pneumothorax or subclavian vein thrombosis)
Physiological	
CSF drainage for surgical field, reduces ICP/brain oedema Carotid/vertebral system unobstructed, reduces ICP	Decreased cerebral blood flow due to lower haemodynamic arterial pressure Venous blood pooling in lower extremities Risk of post-op or tension pneumocephalus (0.2%) [1] Risk of subdural haematoma (1.3%) [11]

### Anaesthetic technique

Routine monitoring includes ECG, continuous end-tidal CO2 (ETCO_2_), oxygen saturations, spirometry, continuous invasive pressure monitoring and right internal jugular vein cannulation (CVC), allowing use of vasopressors, in cardiovascular collapse [8]. Patients are ventilated with an inspired concentration of oxygen (FiO_2_) 40–50%. Indications of potential VAE include hypotension, sudden drop in ETCO_2_ with no change in ventilation settings or drop in oxygen saturations depending on FiO_2_. The neurosurgeon is alerted while the FiO_2_ is increased to 100%, and the surgical field is covered with wet swabs, irrigated with saline. The operating table is tilted head-down to prevent further air entrainment and aid with the VAE-associated hypotension treated with vasopressors and fluid resuscitation. Aspiration of air from the CVC can be performed if its tip is positioned at the superior vena cava/right atrial junction.

### Positioning

**Fig. 2:**
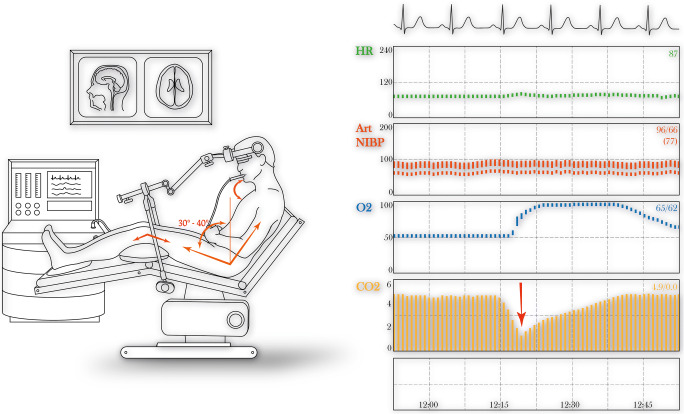
**a** The patient is initially lying completely flat on the operating table and the Mayfield clamp is applied. The sitting position is assumed with the electric controls of the table, gradually, over 1–2 min. The Mayfield clap is secured anteriorly and the superior back support of the table is removed. The head is kept in a perfect midline and the neck is flexed up to 2–3 cm from the jugular notch of the sternum. The legs are elevated by placing pillows under the knees and flexing the hips no more than 45° balancing increase of intrathoracic pressure and lower VAE risk with reduced excursion of the diaphragm and respiratory complications. Excessive hip flexion may also lead to sciatic nerve compression, femoral vein compression under the inguinal ligament and venous pooling and hypotension at the end of the operation. **b** Diagram of a real anaesthetic monitor values, during a sitting position, from our practice. Although a drop ETCO_2_ ≥ 3 mmHg (yellow graph) is often seen, it is not pathognomonic for VAE; visible air bubbles, cardiovascular and haemodynamic instability are also observed in various classification schemes [5] HR, heart rate; Art NIBP, arterial non-invasive blood pressure

The SP is assumed gradually aiming for midline head position with neck flexion (Table 2 and Fig. 2). Neuronavigation can be employed but is of limited assistance, due to depth and proximity of small, key structures.

### Opening

**Fig. 3:**
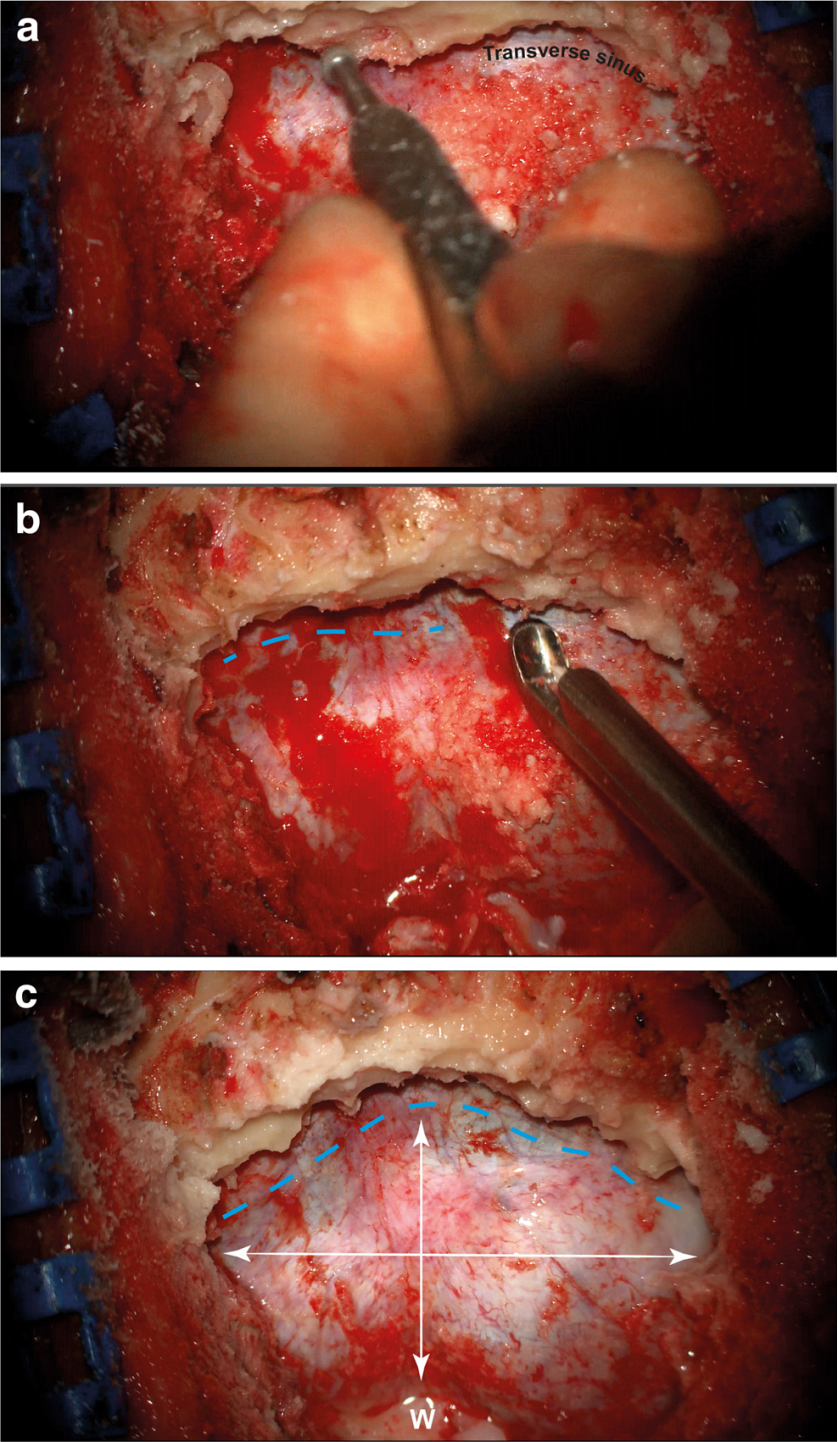
Bony exposure of, at least the inferior half of the transverse sinus and confluence of sinuses, initially with a diamond burr (**a**) and subsequently with Kerrison’s rongeurs (**b**). Adequate lateral and inferior exposure (white arrows, **c**) are essential for the cerebellum to fall down with no retraction

Following a midline muscle dissection from the external occipital protuberance to C2, a craniectomy is performed exposing completely the inferior half of the transverse sinus, initially thinning the bone with a diamond drill and completing the removal with 2-mm Kerrison’s rongeurs. A craniotomy can, alternatively, be performed, provided that the transverse sinus exposure is performed in a controlled and gradual way to avoid inadvertent sinus injury. Adequate lateral and inferior bony removals are critical during exposure allowing wide angle approach and cerebellar fall under gravity, respectively (Fig. 3). The foramen magnum remains intact to avoid cerebellar slump.

### Tumour exposure

**Fig. 4:**
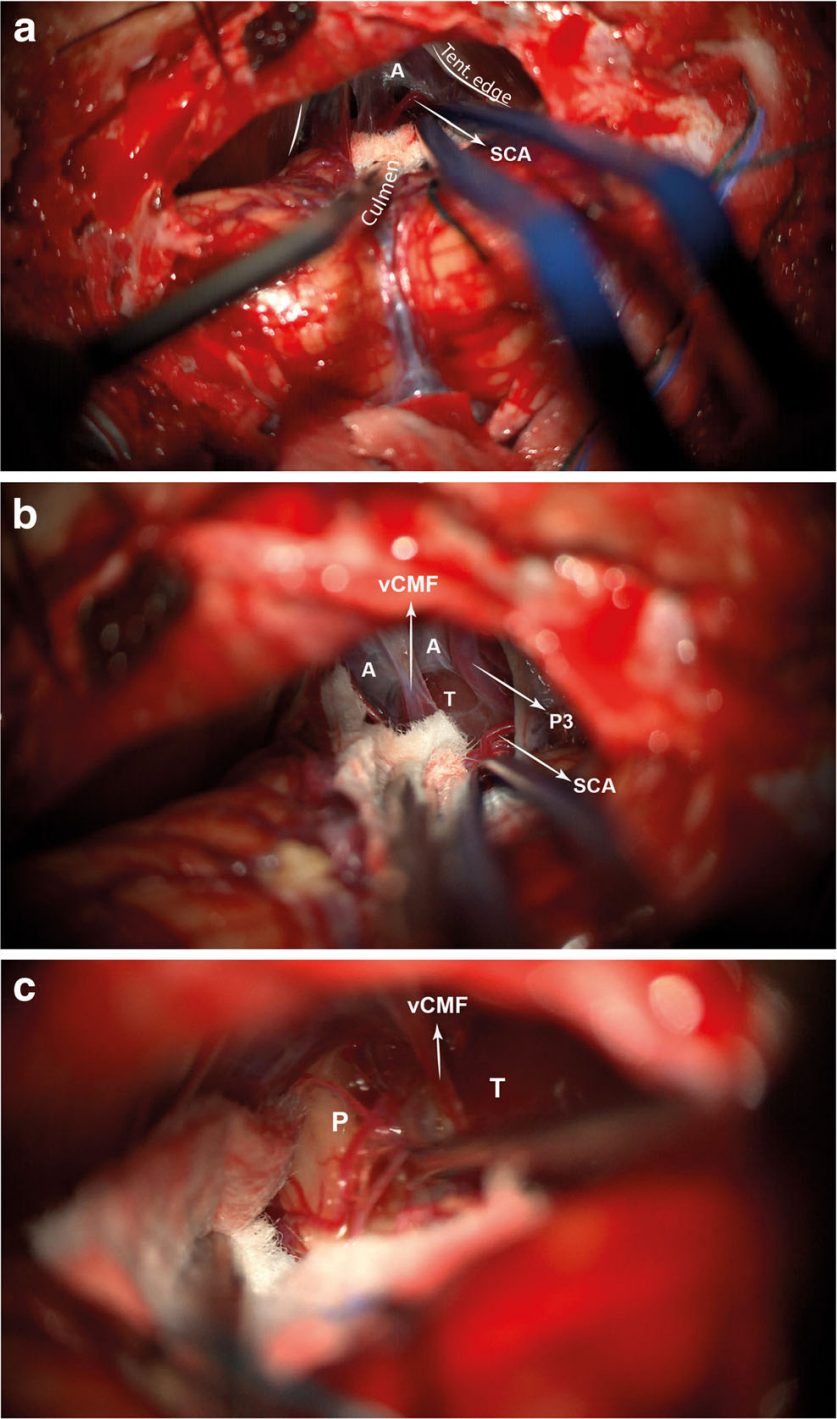
Stepwise exposure of the tumour. The culmen of the vermis is followed inferiorly leading to the tentorial incisura (**a**). The arachnoid membrane, A, is relatively thin in this case. The vein of the cerebello-mesencephalic fissure, vCMF, the third segment of the posterior cerebral artery, P3 and a loop of the superior cerebellar artery, SCA, are seen displaced by the tumour, T (**b**). An initial, exploratory dissection, with Rhoton #8, from the pulvinar of thalamus, P, without sacrificing small arterial branches is performed. T, tumour

The dura is opened in a standard “Y” fashion. Cortical veins suspending the superior cerebellar surface from the tentorium are diathermised at their tentorial attachment, allowing the cerebellum to fall freely, inferiorly. The vermian culmen is followed directly inferiorly until the tentorial edges are identified (Fig. 4a) with an opaque arachnoid membrane stretching over the pineal region, thalami and tectal plate. The thickness of the arachnoid varies according to pathology, from significant to thin (Fig. 4b). Sharp dissection is avoided as the BvRos are directly vulnerable.

### Tumour resection

Prior to any tumour resection, a clear understanding of the surgical field with the position of the vCMF, BvRo, ICv, VG, P3, P4, SCA, superior colliculi and pulvinars (Fig. 4b, c) is required; the pineal gland is not discernible. In addition, the consistency, vascularity, adherence to thalami and tectal plate of the tumour are assessed. The vCMF can be sacrificed in larger tumours.

**Fig. 5:**
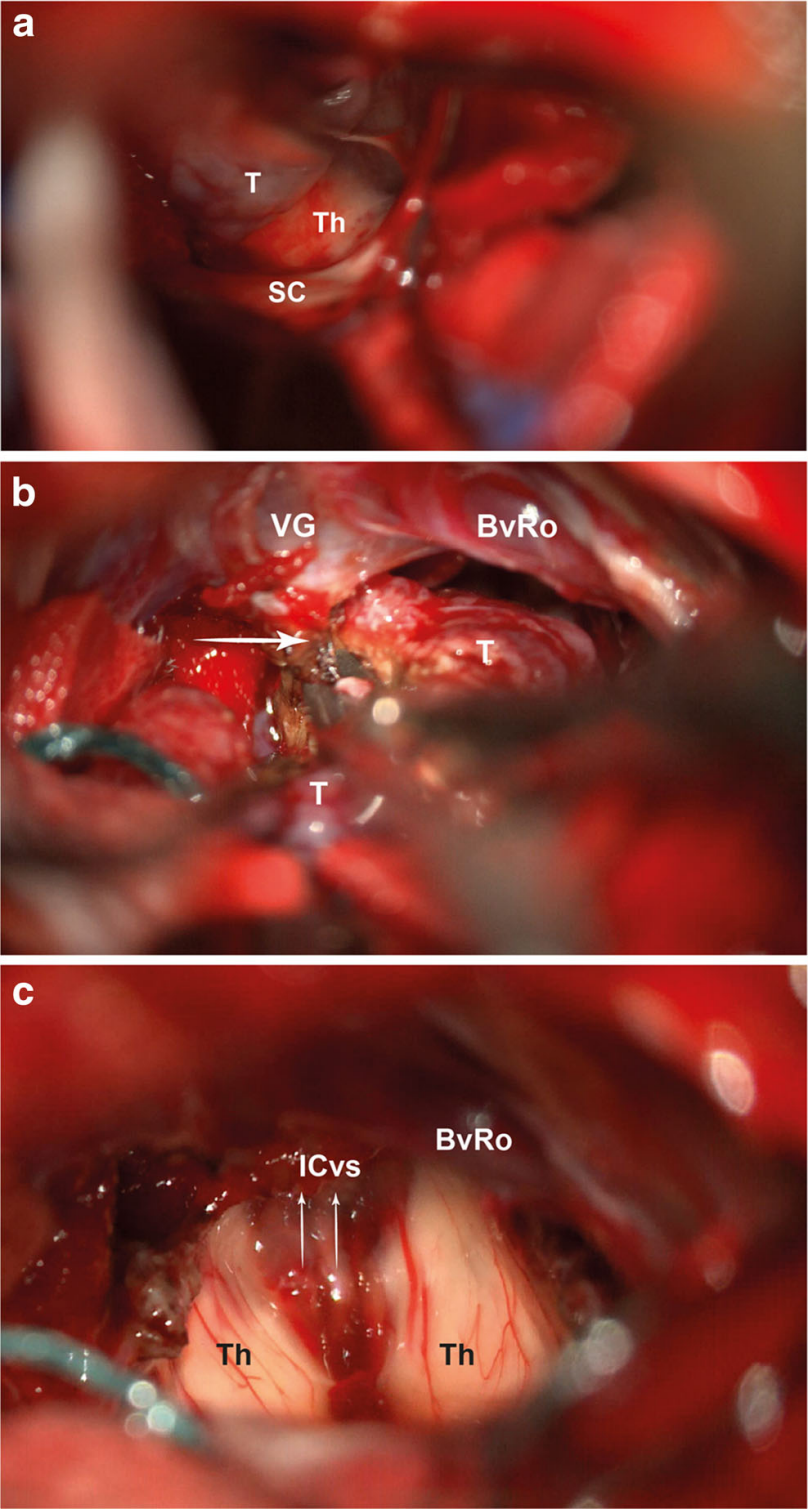
Stages of tumour removal. **a** The tumour is initially dissected laterally from the thalamus, Th, with all traction on the side of the tumour, T. SC, superior colliculus. **b** Following circumferential dissection, the superior attachment is diathermied and incised away from the vein of Galen, VG. BvRo, basal vein of Rosenthal. **c** View of the posterior third vernicle following tumour removal. The ependymal surface and thalami have not been injured during dissection. ICvs, intraventricular part of internal cerebral veins

Although a large tumour can be debulked, our technique is attempting en-bloc removal, as the safest way to distinguish tumoral from normal tissue. A microdissector Rhoton #8 (Integra, Plainsboro, NJ) is used to circumferentially develop a plane with all traction applied to the tumour rather than adjacent brain. The circumferential plane develops easier initially laterally from the pulvinar of the thalami (Fig. 5a), then inferiorly from the tectal plate and finally superiorly from the splenium of the corpus callosum (Fig. 5b).

**Fig. 6:**
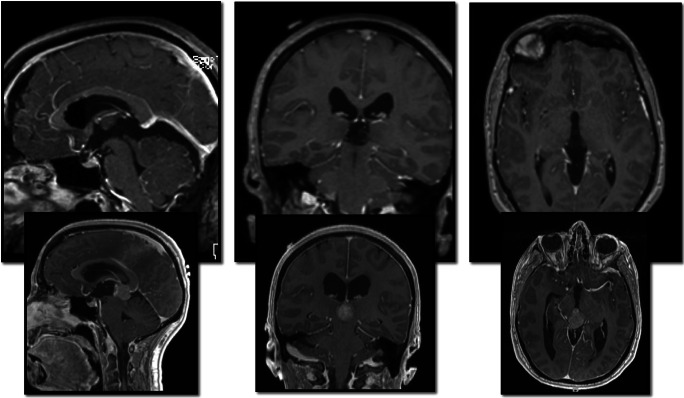
Sagittal, coronal and axial postoperative views (top panel) following tumour resection with preoperative (bottom panel) as reference

No bipolar diathermy is used unless for rare selective vessel coagulation. Once the majority of the tumour is dissected, it can be manipulated as one block revealing its interface and final attachments which are again released with Rhoton #8. The posterior third ventricle is exposed last; the ependymal surfaces remain impeccable with no evidence of manipulation injury (Fig. 5c). A watertight closure with dural patch and dural sealant minimises the risk of CSF leak or pseudomeningocele. Postoperative MRI scan shows complete tumour removal (Fig. 6).

## Indications

Tumours originating from the pineal gland (pineocytomas, pineoblastomas); germ cells (teratoma, embryonal carcinoma, choriocarcinoma); non-germ cell (tectal glioma, falcotentorial meningioma).

## Limitations

Despite the uncommon frequency of these tumours, the IT/SC approach should be undertaken by teams on a relatively regular basis, as anaesthetic and surgical aspects can be challenging.

## How to avoid complications

Carefully planned, layer-by-layer dissection of the pineal region is critical, as major venous injury cannot be repaired and most likely will result in stroke. Sound knowledge of the surgical venous, arterial and neural anatomy of the pineal region is critical.

## Information given to the patient

The patient is consented for systemic complications including VAE, and neurological complications including arterial or venous injury and stroke.

## Supplementary Information

ESM 1 (MP4 431 MB)
